# FMRI activation to cannabis odor cues is altered in individuals at risk for a cannabis use disorder

**DOI:** 10.1002/brb3.1764

**Published:** 2020-08-30

**Authors:** Natalia M. Kleinhans, Julia Sweigert, Matthew Blake, Bradley Douglass, Braden Doane, Fredrick Reitz, Mary Larimer

**Affiliations:** ^1^ Department of Radiology University of Washington Seattle WA USA; ^2^ Integrated Brain Imaging Center University of Washington Seattle WA USA; ^3^ Center on Human Development and Disability University of Washington Seattle WA USA; ^4^ The Werc Shop Bellevue WA USA; ^5^ Department of Psychiatry and Behavioral Sciences University of Washington Seattle WA USA

**Keywords:** addiction, cannabis cues, craving, cue reactivity, marijuana, neuroimaging, reward

## Abstract

**Introduction:**

The smell of cannabis is a cue with universal relevance to cannabis users. However, most cue reactivity imaging studies have solely utilized visual images, auditory imagery scripts, or tactile cues in their experiments. This study introduces a multimodal cue reactivity paradigm that includes picture, odor, and bimodal picture + odor cues.

**Methods:**

Twenty‐eight adults at risk for cannabis use disorder (CUD; defined as at least weekly use and Substance Involvement Score of ≥4 on the Cannabis sub‐test of the Alcohol, Smoking and Substance Involvement Screening Test) and 26 cannabis‐naive controls were exposed to cannabis and floral cues during event‐related fMRI. Between‐group differences in fMRI activation and correlations were tested using FMRIB’s Local Analyses of Mixed Effects and corrected for multiple comparisons using a voxelwise threshold of *z* > 2.3 and a corrected cluster threshold of *p* < .05.

**Results:**

Both visual and olfactory modalities resulted in significant activation of craving and reward systems, with cannabis odor cues eliciting a significantly greater response in regions mediating anticipation and reward (nucleus accumbens, pallidum, putamen, and anterior insular cortex, supplementary motor area, angular gyrus and superior frontal gyrus) and cannabis picture cues eliciting a significantly greater response in the occipital cortex and amygdala. Furthermore, the CUD group showed significantly increased activation in the ventral tegmental area (VTA), the insula, and the pallidum compared to controls. Within the CUD group, activation in the insula, anterior cingulate, and occipital cortex to bimodal cannabis cues was significantly correlated with self‐reported craving.

**Conclusion:**

Our multimodal cue reactivity paradigm is sensitive to neural adaptations associated with problematic cannabis use.

## Graphical abstract

The smell of cannabis is a cue with universal relevance to cannabis users, but this modality is under‐utilized in cue reactivity research. Our fMRI study demonstrated that cannabis odor cues elicit greater activation in reward circuitry than cannabis visual cues.

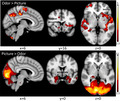

## 1 INTRODUCTION

Craving is fundamentally associated with the transition from recreational drug use to problematic, compulsive drug‐taking behavior (Robinson & Berridge, 1993). Biobehavioral models purport that craving develops through classical conditioning, whereby repeated exposure to environmental cues leads to hypersensitivity to the motivational effects of drugs and drug‐associated stimuli through the neuroadaptation of dopaminergic reward structures (Robinson & Berridge, 1993, 2008). While some individuals can use drugs without becoming addicted, for others this sensitization of reward structures within the dopamine system intensifies ordinary ‘wanting’ into excessive drug craving (Robinson & Berridge, 1993, 2008). Although cannabis has less potential for addiction compared to other substances such as opioids, an estimated 30% of regular cannabis users will become dependent (Hasin et al., 2015).

Everitt and Robbins (2005) state that in the early stages of addiction, a drug is voluntarily taken for its rewarding effects, but a loss of control eventually renders this behavior habitual or compulsive. This shift from voluntary to compulsive behavior is proposed to reflect a transition from prefrontal to striatal neural network control. FMRI studies have provided complementary insight into the neural mechanisms of cannabis craving in humans. Increased activation in response to visual cannabis cues compared to neutral cues has been observed in the ventral tegmental area (VTA), anterior cingulate cortex (ACC), orbital frontal cortex (OFC), striatum, insula, cerebellum, thalamus, pre‐ and postcentral gyri, inferior parietal lobe, and superior temporal gyrus (Cousijn et al., 2012; Filbey, Schacht, Myers, Chavez, & Hutchison, 2009). In addition, activation in the occipital cortex, hippocampal regions, superior temporal pole, and middle occipital gyrus has been shown to be positively correlated with subjective reports of cannabis craving (Charboneau et al., 2013), suggesting these regions may be sensitive to individual differences in addiction severity.

In addition to measures of craving, fMRI activation to cannabis cues has been studied in the context of other problems related to cannabis use. For example, fMRI activation in the nucleus accumbens (NAc) and OFC following exposure to cannabis cues was significantly positively correlated with the Marijuana Problem Scale (Filbey et al., 2009). More recently, a hierarchical linear regression analysis showed that fMRI activation in the putamen at baseline was an independent predictor of the total Cannabis Use Disorder Identification Test (CUDIT) score at a three‐year follow‐up visit, over and above behavioral measures including baseline cannabis use and problem severity, baseline alcohol use and problem severity, baseline nicotine dependence, baseline number of cigarettes per day, baseline craving, and baseline lifetime use of other psychotropic substances (Vingerhoets et al., 2016). Notably, the putamen has a very high expression of CB1R and is adjacent to the pallidum, which has the highest expression of CB1R of all structures implicated in reward and addiction.

Because drug craving often persists (or can resurface) long after drug use has stopped, craving is strongly associated with relapse. cue reactivity has been found to predict treatment outcome and relapse in cigarette, alcohol, and heroin addiction (Grusser et al., 2004; Janes et al., 2010; Marissen et al., 2006; Payne, Smith, Adams, & Diefenbach, 2006), and although limited research has been conducted using cannabis cues, it may also play a role in cannabis use disorders. Understanding factors that contribute to relapse will be critical not only to understanding the process of addiction, but also to developing effective therapies (Jasinska, Stein, Kaiser, Naumer, & Yalachkov, 2014).

Most cannabis cue reactivity studies have utilized pictures, auditory imagery scripts, or tactile cues. While these cues induced craving, there is undoubtedly wide individual variability in their direct relevance across participants. The cues used in experiments are critical, because it is well known that context plays a large role in the expression of sensitization, and individuals with substance use disorders tend to experience craving most strongly when they are in particular drug‐associated contexts (Anagnostaras & Robinson, 1996; Anagnostaras, Schallert, & Robinson, 2002; Robinson, Browman, Crombag, & Badiani, 1998; Stewart & Vezina, 1991). Thus, for example, visual stimuli depicting paraphernalia an individual has never used in a room he/she has never entered may lead to an fMRI brain response that is weaker than would be expected, given the degree of neural sensitization that is neuroanatomically present.

A cannabis paradigm that utilizes a cue with more universal relevance would improve our ability to study the neurobiological basis of craving and its role in the development of addiction and vulnerability to relapse. We propose that capitalizing on the unique odor of cannabis will bring us a step closer to this goal. The behavioral evidence available suggests that olfactory cues in combination with visual, tactile, and/or auditory cues can produce or increase craving. In one study (Haughey, Marshall, Schacht, Louis, & Hutchison, 2008), participants were exposed to a used pipe or bong and asked to focus on it, smell it, and imagine what it would be like to smoke cannabis out of it. Subjective craving following this cue exposure was shown to increase craving over and above the baseline measurement obtained after 5 days of abstinence in daily cannabis users (Haughey et al., 2008). Another study used virtual reality simulations including audio, visual, olfactory, and vibrotactile stimuli. Participants exposed to a “party room” of people smoking cannabis and to a room containing cannabis‐related paraphernalia reported higher drug craving and attention to cannabis‐related cues. Importantly, once they left the cannabis rooms, they returned to baseline in terms of craving/thoughts about smoking (Bordnick et al., 2009). However, neither of these studies were designed to look at the specific role of olfaction, nor at how the integration of multisensory cues modulates the neural craving response.

Our experiment tested a new multimodal cannabis cue reactivity paradigm that included unimodal pictures, unimodal odors, and bimodal cues combining pictures and odors, and examined whether odor stimuli activated mesocorticolimbic regions to a greater degree than picture stimuli. Next, we tested whether our various combinations of cannabis stimulus types and contrasts showed increased activation in mesocorticolimbic regions in CUD participants compared to controls. We hypothesized greater activation to cannabis cues in the CUD group but no significant group difference in response to neutral non‐cannabis (flower) cues on the basis of incentive sensitization theory, positing sensitization as a response to cues predicting drug availability (Robinson & Berridge, 2008). Next, we examined whether higher activation of brain regions involved in craving (prefrontal cortex, anterior cingulate, orbital frontal cortex, hippocampus, and insula) (Koob & Volkow, 2016) was correlated to higher self‐reported craving measured after the cue reactivity fMRI scan. Lastly, we were interested in exploring whether degree of brain activation to cannabis cues would be related to shorter delay in actual use of cannabis in the 24 hr following the end of the cue‐exposure paradigm among those at risk for CUD who are cannabis‐deprived prior to the visit. To test this, participants were contacted the day after their MRI visit and asked about their cannabis use in the past 24 hr.

## 2 MATERIALS AND METHODS

### 2.1 Participants

Twenty‐eight adults at risk for CUD (14 females, 14 males, age range 21–39) and twenty‐six age‐ and sex‐matched cannabis‐naïve adults (control; 12 females, 14 males, age range 21–41) were recruited from the Seattle metropolitan area. All participants were right‐handed. Inclusion in the CUD group was determined using the Cannabis sub‐test of the Alcohol, Smoking and Substance Involvement Screening Test (ASSIST; Humeniuk et al., 2008); participants who qualified as moderate to high risk for a cannabis use disorder (ASSIST Substance Involvement Score ≥ 4) and also reported weekly to daily cannabis use in the prior year were enrolled in the CUD group. Inclusion in the control group was based on self‐report of no lifetime history of cannabis use. Participants in both groups were additionally screened using a semi‐structured interview and excluded for the following: received a diagnosis of or received treatment for schizophrenia or other psychotic disorder, bipolar disorder, or depression within the past 6 months; reported high risk alcohol use (CAGE score > 2), or reported Moderate to High Risk use of other illicit substances (ASSIST Substance Involvement Score ≥ 4 for each substance reported, e.g., inhalants, cocaine); or reported current psychotropic medication, significant neurological medical history, clinically diagnosed hyposmia or anosmia, MRI contraindications, or left‐handedness. Details on illicit substance use in our participants are included in the Supporting information section. Current tobacco use was not an exclusionary criterion for enrollment in either group and therefore was not assessed during screening. Following data acquisition, two participants (1 CUD, 1 control) were excluded from analyses due to subsequent report of psychotropic medication use, one CUD participant was excluded because his permanent retainer caused severe signal drop‐off, and another CUD participant was excluded because of technical issues with the olfactometer. The final sample included 25 controls and 25 CUD participants.

### 2.2 Procedures

The following study procedures were approved by the University of Washington Human Subjects Division Institutional Review Board and informed written consent was obtained from all participants. Participants were compensated for their participation, receiving $50 at the end of the research visit and an additional $25 after completing the follow‐up phone interview one day later.

### 2.3 Substance use questionnaires

All participants were asked to refrain from using cannabis for at least 48 hr prior to the research visit. Three CUD participants (out of 25) abstained from cannabis use for less than 48 hr (34.35 hr, 46.06 hr, 47.60 hr).

Before entering the scanner, participants were screened to ensure they did not exhibit symptoms of upper airway breathing disorders or acute cold symptoms. Then, they were asked to complete questionnaires pertaining to current and prior use of substances, specifically of cannabis, alcohol, and tobacco. The Marijuana Craving Questionnaire—Short Form (MCQ‐SF) provided a measure of participants’ level of self‐reported craving for cannabis at the time of the research visit (Heishman et al., 2009). The Cannabis Use Disorder Identification Test—Revised (CUDIT‐R) was used to assess severity of problematic cannabis‐related behaviors (Adamson et al., 2010). Detailed information was also collected regarding participants’ individual histories of cannabis use, including age of first use, frequency of use, and duration of frequent use (total number of years with at least weekly use). The Tobacco sub‐test of the ASSIST was used to collect information on concurrent tobacco use (Humeniuk et al., 2008). The Alcohol Use Disorder Identification Test (AUDIT) was administered to quantify participants’ alcohol use and dependence symptoms (Saunders, Aasland, Babor, de la Fuente, & Grant, 1993). In a follow‐up phone call approximately 24 hr after the end of the MRI visit, participants were asked: (a) “Have you used marijuana since you had your brain scan?” (b) “What time did you begin using marijuana?” (c) “How much marijuana have you used since you had your brain scan at [time the participant's scan ended]?” Participants were notified in advance that they would be receiving a follow‐up phone call but were not given advance information about the content of the phone call to avoid influencing their post‐visit behavior. A full summary of participants’ demographic information and substance use measures is available in Table 1; additional details about participants’ patterns and quantities of cannabis use are available in Supporting information.

**Table 1:** Participant characteristics

	CUD (*n* = 25)	Control (*n* = 25)
Sex (M:F)	13:12	13:12
Race		
Caucasian	21	12
Asian	1	11
African American	1	0
Other	2	2
Ethnicity		
Hispanic/Latino	3	1
Not Hispanic/Latino	22	24

	Mean*SD*		Mean*SD*		*p*
Age (years)	26.17	(4.15)	26.21	(5.05)	.981
AUDIT	6.6	(4.33)	2.52	(2.12)	<.001
ASSIST—Tobacco	3.46	(5.59)	0.56	(1.89)	.012
CUDIT‐R	10.36	(5.19)	0.00	(0.00)	<.001
MCQ‐SF	34.16	(9.13)	17.08	9.53	<.001
Age at first use of cannabis (years)	17.32	(4.66)			

	*n*	%
Mode of cannabis consumption		
Cannabis “Bud”	24	96
Extract (concentrated oil, wax, etc.)	9	36
Edibles	14	56
Hash	3	12
Frequency of cannabis use		
Monthly or less	0	0
2–4 times per month	5	20
2–3 times per week	8	32
4+ times per week	12	48

	Mean	*SD*	Range
Grams (g.) of cannabis per typical day among “bud” users (*n* = 24)	0.37	0.32	0.05–1.0 g
Milligrams (mg.) of THC per typical day among edible users (*n* = 10)	17.15	13.46	4.0–40.0 mg
Milligrams (mg.) of CBD per typical day among edible users (*n* = 5)	10.50	2.74	7.5–15.0 mg
Duration of at least weekly use of cannabis (years)	4.07	3.32	0.75–11.5 years
Time between last cannabis use and MRI scan (days)	4.10	3.39	1.43–13.8 days

Abbreviations: ASSIST, Alcohol, Smoking and Substance Involvement Screening Test; AUDIT, Alcohol Use Disorder Identification Test; CBD, Cannabidiol; CUDIT‐R, Cannabis Use Disorder Identification Test—Revised; *SD*, Standard Deviation; THC, Tetrahydrocannabinol.

### 2.4 fMRI scan

A fast event‐related fMRI scan, adapted from Gottfried and Dolan (2003), was collected as part of a longer imaging protocol. Before and after the beginning of the fMRI task, participants were administered the Visual Analog Scales (VAS) over the intercom and asked to rate how much they agreed with the VAS statements on a scale from 1 to 10. The VAS statements were: (a) “I *crave* marijuana right now.” (b) “I *want* to use marijuana right now.” (c) “Marijuana sounds very *appealing* to me right now.” After the participants exited the scanner (approximately 15 min after completing the fMRI task), they were asked to rate how pleasant they found the cannabis odorant (range of −3 unpleasant to 3 pleasant) and how much the odorant smelled like cannabis (range of 0 = not at all to 6 = immediately recognizable).

During the fMRI task, participants were exposed to unimodal and bimodal stimuli that included pictures of cannabis products/paraphernalia, pictures of nonpsychoactive garden‐variety flowers and related products, the cannabis odorant Cannaroma® (created by The Werc Shop™, based on the terpene profile of the cannabis strain “Blue Dream”), and pure phenylethyl alcohol (concentration ≥ 99% v/v; Sigma‐Aldrich), which smells like roses. 0.05 ml of each odorant was dropped onto a 1‐inch diameter filter paper and placed in two separate odorant chambers of the olfactometer.

Air moved continuously through the Constant Flow path at a rate of 0.5 L/min via a normally open solenoid valve (Cole‐Parmer #EW‐01540‐09) to the nosepiece for the length of the entire fMRI protocol. The Odor/No Odor pathway used a 6‐in‐1‐out solenoid manifold (Cole‐Parmer #RK‐01356‐16), driven by a USB‐controlled relay array (Measurement Computing SwitchAndSense‐8/8) directed by our custom LabVIEW™ software, to send air at 1.25 L/min through either the Control Flow (no odor) path during the ITI, the cannabis odorant‐containing chamber, or the PEA odorant‐containing chamber for each two‐second trial. The Constant Flow, Control Flow, and Odorant pathways merged at the nosepiece delivery tube, which was mounted on the head coil adjacent to the participant's nose, which was located approximately 125 mm away routed through 1/8″ in diameter tubing. Care was taken to verify that the flow rate did not differ upon switching between the control and odorant paths to within the precision of the flowmeters when tested with minimal (<1 m) tubing lengths. The total flow rate was 1.75 L/m, and the steepness of stimulus onset was 30 ms.

**Figure 1:**
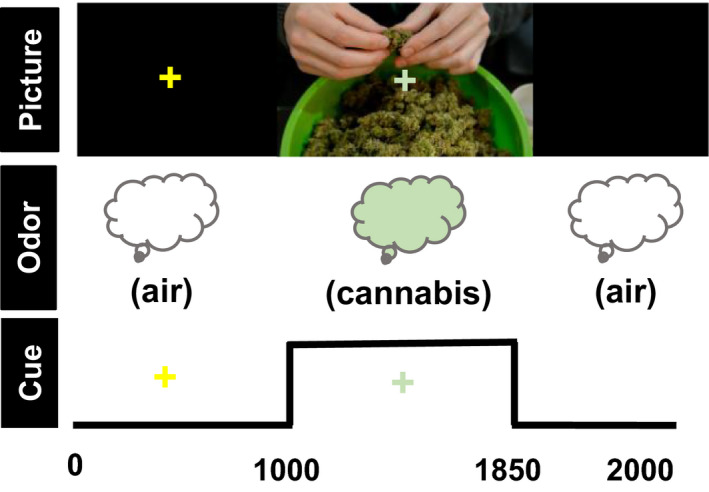
Example bimodal cannabis trial with stimulus timing (ms) information. Participants were instructed to *prepare* to breathe in when they saw the yellow cross and to *inhale* when they saw the green cross

**Figure 2:**
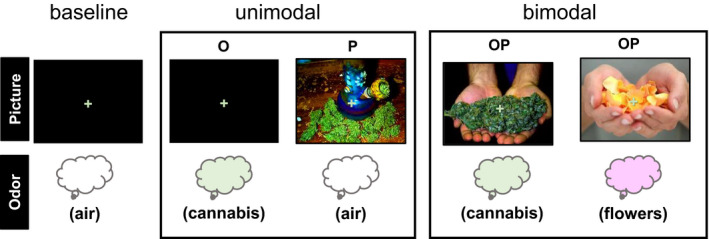
Example stimuli for each trial type. O = odor, P = picture; OP = simultaneous odor and picture stimuli

Stimuli were presented with Presentation™. Participants were instructed to prepare to breathe in when they saw a yellow cross‐hair and to inhale through their nose when the green cross‐hair appeared. They were informed that sometimes they would be able to smell a flowery smell, sometimes they would smell a cannabis‐like smell, and sometimes they would smell neither. They were also informed that there was no THC in the cannabis odorant (Faria, Han, Joshi, Enck, & Hummel, 2020). During each 2‐s trial, a 1,000 ms yellow warning cue appeared, signaling the participant to pause their breathing and prepare to sniff once the green cross‐hair appeared. The green cross‐hair was presented in synchrony with the 850 ms baseline stimuli (plain air; *n* = 22), odor stimuli (O; *n* = 44) or picture stimuli (P; *n* = 44) or odor + picture stimuli (OP; *n* = 44), followed by a 250 ms interstimulus interval. A sniff was made on every trial, regardless of odorant presence. See Figure 1 for an example OP cannabis trial. Within each stimulus modality, half the trials presented cannabis images and/or odors and the other half presented flower images and/or odors. See Figure 2 for example stimuli. Participants were also exposed to incongruent OP stimuli (e.g., cannabis odor paired with a picture of roses). Incongruent trials were not included in this report. The order of trials was optimized using optseq2 (https://surfer.nmr.mgh.harvard.edu/optseq/) and the experiment lasted 12 min and 10 s.

### 2.5 Olfactometer details

The olfactometer used in this experiment was built in the Instrument Development Laboratory at the University of Washington Center on Human Development (see Kleinhans et al., 2018, for more detail). The olfactometer design was based on Lorig, Elmes, Zald, and Pardo (1999), with a modification of the odorant cylinders/manifold and nosepiece. The intent of the olfactometer design was to allow rapid switching between olfactory stimuli without interrupting the flow of air. By locating the solenoid valves outside of the scanner room, the design also ensures that participants do not receive any auditory cues to indicate the changing olfactory stimulus. 0.05 ml of each odorant was dropped onto 1‐inch diameter filter papers and placed in their respective odorant chamber of the olfactometer. Participants were not exposed to the odorants prior to the fMRI task.

#### 2.5.1 MR data acquisition

Structural and functional MRI data were acquired on a Philips Achieva 3.0T scanner (Version 1.5, Philips Medical Systems) with Quasar Dual gradients, using a 32‐channel SENSE head coil. A T1‐weighted 3D MPRAGE (magnetization‐prepared rapid gradient echo; TR = 7.6 ms, TE = 3.6 ms, inversion delay (TI) = 910.5 ms, flip angle = 7°, field of view = 256 × 256 × 176 mm^3^, 176 × 256 matrix, voxel size = 1 × 1 × 1 mm^3^, TFE shots = 128, TFE durations = 1,963.3 ms, REST slab 64.2 mm slice thickness, with 176 slices acquired in transverse slice orientation with fold‐over in the anterior–posterior direction) was collected for co‐registration and anatomical localization. fMRI data were acquired with the following parameters: TR = 2,000 ms, TE = 24 ms, flip angle = 79°, field of view = 240 × 240 × 156 mm^3^, matrix = 80 × 78, voxel size = 3 × 3 × 4 mm^3^, 39 slices with 0‐mm gap, and a total acquisition time of 12 min, 10 s.

### 2.6 Data processing

MRI data preprocessing was performed using FSL (http://www.fmrib.ox.ac.uk/fsl/), AFNI (http://afni.nimh.nih.gov/afni/) and ANTS (https://www.nitrc.org/projects/ants/). Our preprocessing pipeline consisted of (a) motion correction, (b) spike artifact removal, (c) high‐pass filtering [sigma = 50 s], and (d) spatial smoothing [FWHM = 5 mm]. The mean time series of cerebral spinal fluid from the ventricles was extracted, and along with rigid body motion parameters and single‐point motion regressors (framewise displacement and dvars calculated via fsl_motion_outliers), were included as nuisance regressors. To reduce the effects of head motion, a mean absolute motion value (RMS, as calculated by FSL mcflirt) >1.0 was set as the exclusion threshold. None of our participants exceeded this motion threshold.

Time series analyses were carried out using FILM with local autocorrelation correction (Woolrich, Ripley, Brady, & Smith, 2001). Regressors were defined to model activity during the following 850 ms stimulus cues: cannabis odor, flower odor, cannabis picture, flower picture, cannabis odor + picture, and flower odor + picture. These regressors were convolved using a single gamma function to account for hemodynamic lags. Condition effects were estimated at each voxel yielding the following contrasts for each participant: cannabis odor > baseline, cannabis picture > baseline, cannabis odor + picture>baseline, cannabis odor > flower odor, flower odor > cannabis odor, cannabis picture > flower picture, flower picture > cannabis picture, cannabis odor > cannabis picture, cannabis picture > cannabis odor, and cannabis odor + picture>flower odor + picture. FMRI data were registered to the MPRAGE and then warped to MNI template brain via ANTS diffeomorphic registration (Avants et al., 2011).

Higher‐level analyses were carried out using FLAME (FSL's Local Analysis of Mixed Effects) stage 1 and stage 2 (Beckmann, Jenkinson, & Smith, 2003). Whole‐brain voxelwise comparisons of fMRI activation values in CUD and control participants were performed using *t*‐ and *F*‐statistics, which were then converted to *z*‐scores by means of a probability integral transformation and thresholded using clusters determined by *z* ≥ 2.3 and a (corrected) cluster significance threshold of *p* < .05 (Woolrich, Behrens, Beckmann, Jenkinson, & Smith, 2004). Additional a‐priori small volume region of interest analyses were conducted in the right and left nucleus accumbens, right and left pallidum, and the VTA. Correlations between the crave VAS rating acquired after the fMRI task and activation to cannabis cues were also conducted in the CUD group, and thresholded using clusters determined by *z* ≥ 2.3 and a (corrected) cluster significance threshold of *p* < .05 (Poline, Worsley, Evans, & Friston, 1997).

## 3 RESULTS

### 3.1 VAS rating

Visual Analog Scales ratings were collected before and after the fMRI cue reactivity tasks. Independent samples *t* tests were used to analyze group differences on the VAS. For the ratings collected before the fMRI scan, the average “crave” rating scores for the control group was *M* = 1, *SD* = 0 and for the CUD group *M* = 3.12, *SD* = 2.15, *p* < .001; the average “want” score for the control group was *M* = 1, *SD* = 0 and for the CUD group *M* = 3.68, *SD* = 2.36, *p* < .001; the average “appeal” score for the control group was *M* = 1.24, *SD* = 0.83 and for the CUD group *M* = 4.36, *SD* = 2.94, *p* < .001. For the rating collected after the fMRI scan, the average “crave” score for the control group was *M* = 1.04, *SD* = 0.2 and for the CUD group *M* = 3.6, *SD* = 2.48, with *p *< .001; the average “want” score for the control group was *M* = 1.08, *SD* = 0.28 and for the CUD group *M* = 3.92, *SD* = 2.87, *p* < .001; the average “appeal” score for the control group was *M* = 1.28, *SD* = 1.02 and for the CUD group *M* = 4.64, *SD* = 3.09, *p* < .001. Within the CUD group, there was a statistically significant increase in self‐reported craving following exposure to the cannabis cues (*p* = .045, Friedman test) but not in self‐reported wanting (*p* = .617, Friedman test) or in the appeal of cannabis (*p* = .285, Friedman test).

### 3.2 Post‐scan cannabis use

Cannabis use disorder participants were queried regarding their cannabis use within the first 24 hr after leaving the research visit. Of the 25 included CUD participants, 13 reported using cannabis within 24 hr of the study visit. Of those participants who reported using cannabis, the mean time interval between the end of the research visit and subsequent cannabis use was 330.92 min (*SD* = 352.78 min; range 41–1,275 min). The time of day appeared to be an important factor. The median and mode time of the day the use occurred was 9:00 p.m., and the mean time of the day the use occurred was 8:34 p.m. (range = 3 p.m. to 11:30 p.m.). Two participants used cannabis within an hour after completing the experiment. Of these, one participant finished the MRI at 7:05 p.m. and used cannabis at 8 p.m.; the other finished the MRI at 6:08 p.m. and used cannabis at 7:00 p.m. Further, the correlation between time interval and the post‐fMRI VAS crave score for those individuals who did consume (*n* = 13) was not significant (*r* = −.29, *p* > .05; with one outlier removed *r* = −.09). This suggests that while the cue reactivity paradigm did increase self‐reported craving during the scan, this elicited craving probably did not persist after the experiment was completed.

### 3.3 FMRI motion analyses

An independent samples *t* test was conducted to assess head motion during fMRI acquisition. No significant group differences were found. The absolute root mean square for the CUD group was *M* = 0.332, *SD* = 0.140 and for the control group was *M* = 0.323, *SD* = 0.128, with *p *= .820.

### 3.4 FMRI cue reactivity in the CUD group

#### 3.4.1 Unimodal cues

**Figure 3:**
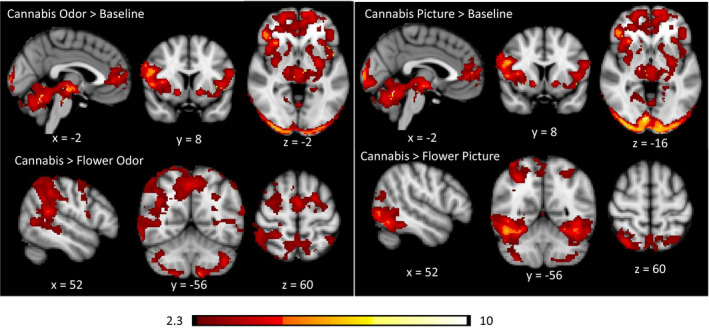
Significant CUD group average cluster maps for unimodal activation trial contrasts. Odor contrasts are on the left panel, and picture contrasts are on the right panel. Data are presented in radiological convention (R = L). Coordinates are in MNI space. The color bar represents *z* values

Group activation maps for the CUD group were similar for unimodal visual and olfactory cannabis cues compared to the baseline condition. Both cue types activated the bilateral prefrontal cortex, insular cortex, nucleus accumbens, striatum, thalamus, VTA, substantial nigra, cerebellum, and occipital lobe (Figure 3).

When the flower cues were included as the control condition, the activation maps differed considerably from when baseline was modeled as the control condition (Figure 3). For the odor cues (cannabis odor > flower odor), significantly greater activation was observed in the precuneus, the frontal eye fields, the supplementary motor area, superior parietal lobes, angular gyrus, supramarginal gyrus, middle temporal gyrus, right striatum, right thalamus, and various regions in the cerebellum (e.g., crus I, crus II, VIIIa, VIIb). For the visual cues, significantly greater activation was observed in the lateral occipital cortex, inferior temporal gyrus, thalamus, and various regions of the cerebellum (e.g., crus I, crus II, VIIIa, VIIb, vermis VI).

**Figure 4:**
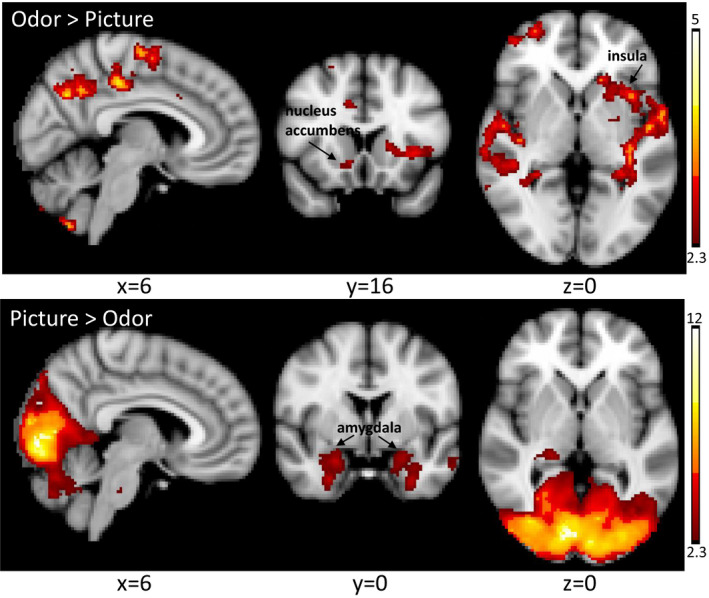
Brain activation to cannabis cues differs according to cue modality in CUD participants. Data are presented in radiological convention (R = L). Coordinates are MNI space. Color bar represents *z* values

The direct comparison of cannabis odor cues to cannabis pictures cues elicited different patterns of activation to each cue modality (Figure 4). The odor cues yielded greater activation in the angular gyrus, supramarginal gyrus, supplementary motor cortex, superior frontal gyrus, left putamen, left pallidum, left insula, right nucleus accumbens (ROI analysis only), posterior cingulate, precuneus, and superior temporal gyrus. The visual cues yielded greater activation in the entire occipital lobe, bilateral amygdala, inferior temporal gyrus, orbital frontal cortex, thalamus, left frontal pole, pons, and cerebellum. Activation in the VTA was not modulated by stimulus modality.

#### 3.4.2 Bimodal cues

**Figure 5:**
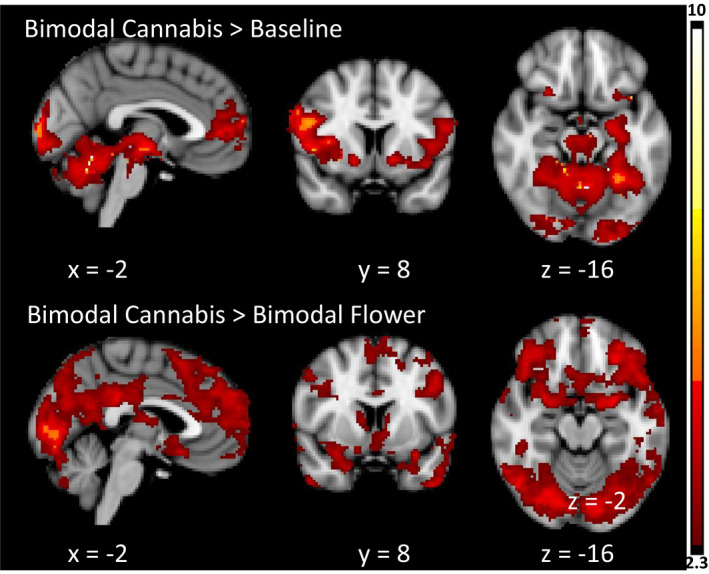
Significant CUD group average cluster maps for bimodal activation trial contrasts. Data are presented in radiological convention (R = L). Coordinates are in MNI space. Color bar represents *z* values

Cannabis use disorder group activation maps for the bimodal cue condition compared to baseline were spatially similar to the unimodal olfactory cue compared to baseline. Activation included the bilateral prefrontal cortex, insular cortex, nucleus accumbens, striatum, thalamus, VTA, substantial nigra, cerebellum, and occipital lobe. When the bimodal flower cue condition was used as the control condition, significant activation was observed in the orbitofrontal cortex, dorsolateral prefrontal cortex, medial prefrontal cortex, cingulate cortex, posterior cingulate and precuneus, occipital cortex, temporal occipital fusiform cortex, caudate, and cerebellum (Figure 5).

A detailed report of the significant activation can be found in Table 2.

**Table 2:** fMRI activation to cannabis cues in the CUD group

Contrast	Voxels	*p*‐val	*z*‐max	*x* (mm)	*y* (mm)	*z* (mm)	Peak region	Other regions
Cannabis odor > Baseline	33,360	<.001	14.2	6	−62	−26	Vermis VI	Ventral Tegmental Area—VTA; Left VI; Right V; Frontal Pole; Left V; Right I‐IV; Left I‐IV; Right VI; Left Crus I; Right Crus I; Left Substantia Nigra; Right Substantia Nigra; Occipital Pole; Lateral Occipital Cortex—superior division; Left Crus II; Brain‐Stem; Paracingulate Gyrus; Right Crus II; Insular Cortex; Lateral Occipital Cortex—inferior division; Right Putamen; Vermis VIIIa; Left Putamen; Central Opercular Cortex; Cingulate Gyrus—anterior division; Temporal Occipital Fusiform Cortex; Lingual Gyrus; Precentral Gyrus; Left Hippocampus; Inferior Frontal Gyrus—pars opercularis; Vermis Crus II; Right Thalamus; Frontal Operculum Cortex; Inferior Frontal Gyrus—pars triangularis; Vermis IX; Left Amygdala; Vermis VIIIb; Left Thalamus; Temporal Fusiform Cortex—posterior division; Left Pallidum; Parahippocampal Gyrus—posterior division; Left VIIb; Frontal Orbital Cortex; Angular Gyrus; Vermis VIIb; Vermis X; Left VIIIa; Right Caudate; Left Caudate; Right Pallidum; Right VIIb; Left Accumbens; Left VIIIb; Right Accumbens; Vermis Crus I; Right Amygdala; Left IX; Right VIIIa; Right IX
Cannabis picture > Baseline	36,866	<.001	13.5	2	−56	−16	Right V	Ventral Tegmental Area—VTA; Left VI; Left V; Right I‐IV; Right VI; Frontal Pole; Left I‐IV; Left Crus I; Right Crus I; Left Substantia Nigra; Occipital Pole; Right Substantia Nigra; Vermis VI; Lateral Occipital Cortex—superior division; Left Crus II; Lateral Occipital Cortex—inferior division; Brain‐Stem; Right Crus II; Paracingulate Gyrus; Lingual Gyrus; Insular Cortex; Vermis VIIIa; Right Putamen; Left Putamen; Temporal Occipital Fusiform Cortex; Central Opercular Cortex; Cingulate Gyrus—anterior division; Vermis IX; Left Hippocampus; Inferior Frontal Gyrus—pars opercularis; Vermis Crus II; Precentral Gyrus; Vermis VIIIb; Inferior Frontal Gyrus—pars triangularis; Right Thalamus; Frontal Operculum Cortex; Occipital Fusiform Gyrus; Temporal Fusiform Cortex—posterior division; Left Amygdala; Left Thalamus; Parahippocampal Gyrus—posterior division; Left VIIb; Vermis X; Left Pallidum; Frontal Orbital Cortex; Vermis VIIb; Left VIIIa; Right VIIb; Left Caudate; Right Pallidum; Left VIIIb; Left IX; Left Accumbens; Right Caudate; Vermis Crus I; Right Amygdala; Right VIIIa; Right Accumbens; Right IX
Cannabis odor + Picture > Baseline	37,645	<.001	14.5	2	−62	−26	Vermis VI	Ventral Tegmental Area—VTA; Left VI; Right V; Left V; Frontal Pole; Right VI; Right I‐IV; Left I‐IV; Left Crus I; Right Crus I; Left Substantia Nigra; Occipital Pole; Right Substantia Nigra; Lateral Occipital Cortex—superior division; Left Crus II; Lateral Occipital Cortex—inferior division; Right Crus II; Brain‐Stem; Paracingulate Gyrus; Insular Cortex; Lingual Gyrus; Vermis VIIIa; Right Putamen; Left Putamen; Central Opercular Cortex; Temporal Occipital Fusiform Cortex; Cingulate Gyrus—anterior division; Vermis IX; Left Hippocampus; Inferior Frontal Gyrus—pars opercularis; Vermis Crus II; Precentral Gyrus; Inferior Frontal Gyrus—pars triangularis; Frontal Operculum Cortex; Vermis VIIIb; Left Amygdala; Temporal Fusiform Cortex—posterior division; Right Thalamus; Occipital Fusiform Gyrus; Left Thalamus; Parahippocampal Gyrus—posterior division; Frontal Orbital Cortex; Left VIIb; Left Pallidum; Vermis X; Vermis VIIb; Left Caudate; Left VIIIa; Right Pallidum; Left VIIIb; Right VIIb; Left IX; Left Accumbens; Right Caudate; Right Amygdala; Vermis Crus I; Right Accumbens; Right VIIIa; Right IX
Cannabis odor > Flower odor	3,789	<.001	6.81	−20	−78	−52	Left VIIb	Brain‐Stem; Left Crus II; Left Crus I; Left VIIIa; Right VIIIa; Left VIIIb; Right VIIb; Right Crus II; Right VIIIb; Left IX; Right Crus I; Left VI; Right IX; Right VI; Left X; Vermis VIIIb; Vermis IX; Vermis Crus II; Vermis VIIIa
	23,046	<.001	5.49	−64	−40	28	Supramarginal Gyrus—anterior division	Frontal Pole; Precuneus Cortex; Juxtapositional Lobule Cortex; Supramarginal Gyrus—posterior division; Parietal Operculum Cortex; Right Thalamus; Lateral Occipital Cortex—inferior division; Planum Temporale; Cingulate Gyrus—posterior division; Cingulate Gyrus—anterior division; Precentral Gyrus; Angular Gyrus; Postcentral Gyrus; Middle Temporal Gyrus—temporooccipital part; Lateral Occipital Cortex—superior division; Frontal Operculum Cortex; Superior Parietal Lobule; Right Caudate; Left Thalamus; Right Putamen; Right Hippocampus
Cannabis picture > Flower picture	1,118	<.001	3.82	14	−34	−2	Right Thalamus	Left Thalamus; Cingulate Gyrus—posterior division; Parahippocampal Gyrus—posterior division; Right Hippocampus; Lingual Gyrus; Precuneus Cortex; Right Caudate; Brain‐Stem; Right Pallidum
	25,647	<.001	6.53	46	−60	−18	Temporal Occipital Fusiform Cortex	Left Crus I; Right Crus I; Left VI; Right VI; Left Crus II; Lateral Occipital Cortex—superior division; Lateral Occipital Cortex—inferior division; Left VIIb; Right Crus II; Right VIIb; Left VIIIa; Occipital Pole; Right VIIIa; Left VIIIb; Occipital Fusiform Gyrus; Vermis VI; Temporal Fusiform Cortex—posterior division; Inferior Temporal Gyrus—temporooccipital part; Right VIIIb; Right V; Left IX; Lingual Gyrus; Left V; Precuneus Cortex; Parahippocampal Gyrus—posterior division; Vermis Crus II; Middle Temporal Gyrus—temporooccipital part; Frontal Orbital Cortex; Intracalcarine Cortex; Left X; Right X; Temporal Fusiform Cortex—anterior division; Right IX; Right Hippocampus; Right Amygdala; Vermis VIIIb; Vermis IX; Vermis VIIIa; Right I‐IV; Left Hippocampus; Left I‐IV; Vermis Crus I; Brain‐Stem; Vermis VIIb
Cannabis bimodal > Flower bimodal	8,432	<.001	4.96	−22	30	38	Superior Frontal Gyrus	Frontal Pole; Paracingulate Gyrus; Cingulate Gyrus—anterior division; Middle Frontal Gyrus; Precentral Gyrus; Frontal Medial Cortex; Juxtapositional Lobule Cortex; Inferior Frontal Gyrus—pars opercularis
	35,723	<.001	6.43	−10	−90	0	Occipital Pole	Right Crus I; Left Crus I; Right VI; Lateral Occipital Cortex—superior division; Left VI; Right Crus II; Left Crus II; Frontal Orbital Cortex; Temporal Pole; Precuneus Cortex; Cingulate Gyrus—posterior division; Lateral Occipital Cortex—inferior division; Lingual Gyrus; Vermis VI; Left VIIb; Occipital Fusiform Gyrus; Middle Temporal Gyrus—posterior division; Temporal Fusiform Cortex—posterior division; Intracalcarine Cortex; Frontal Pole; Right VIIb; Temporal Occipital Fusiform Cortex; Inferior Temporal Gyrus—temporooccipital part; Subcallosal Cortex; Cuneal Cortex; Angular Gyrus; Vermis Crus II; Right Amygdala; Parahippocampal Gyrus—anterior division; Left V; Right V; Middle Temporal Gyrus—temporooccipital part; Right Caudate; Left Amygdala; Right VIIIa; Middle Temporal Gyrus—anterior division; Right I‐IV; Superior Temporal Gyrus—posterior division; Left Thalamus; Right Thalamus; Vermis VIIIa; Left Caudate; Left Hippocampus; Left I‐IV; Left VIIIa; Left Accumbens; Vermis Crus I; Right Hippocampus; Right Accumbens; Vermis VIIb; Left Putamen; Right Putamen; Brain‐Stem; Right VIIIb; Vermis VIIIb

Note

R = right, L = left. *x*, *y*, and *z* coordinates are in Montreal Neurological Institute space. "Peak region" and "other regions" are labeled using the Harvard‐Oxford Cortical Structural Atlas, the Harvard‐Oxford Subcortical Structural Atlas, the Cerebellar Atlas, and the Duke Midbrain Atlas. *p*‐values are based on a whole‐brain cluster correction for multiple comparisons.

### 3.5 Group differences

#### 3.5.1 Unimodal cues

**Figure 6:**
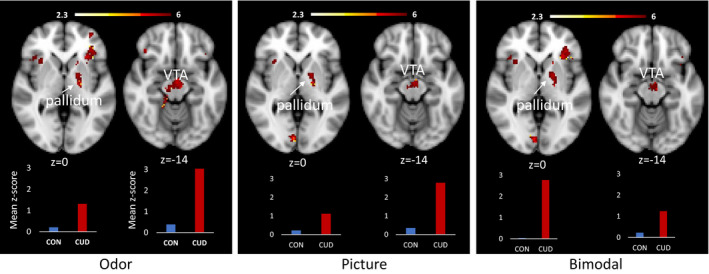
Significant between‐group differences in fMRI activation to unimodal and bimodal cannabis cues compared to baseline. The clusters depicted reflect significant activation based on Whole‐brain correction (insular cortex, VTA [odor only], and occipital cortex) and region of interest analyses (VTA [picture and bimodal], and pallidum). Bar graphs show the mean *z*‐values for all voxels within the significant pallidum (left) and VTA (right) cluster for the CON group (blue) and the CUD group (red). Images are presented in radiological convention (R = L). Coordinates are MNI space. The color bar represents *z* values for the fMRI activation clusters

The CUD group showed greater activation than the control group for the cannabis odor > baseline in the VTA, substantia nigra, frontal pole, striatum, and bilateral insula. The region of interest analyses yielded significant activation in the left pallidum (*p* = .0159), but not the nucleus accumbens (*p* > .05). The cannabis pictures > baseline analysis showed a similar pattern, with significantly greater activation in the right insular cortex and the intracalcarine cortex. The region of interest analysis yielded significant activation in the VTA (*p* = .0114) and the left pallidum (*p* = .0216), but not the nucleus accumbens (*p* > .05). The contrasts that used the flower cue as the control condition, (cannabis odor > flower odor and cannabis picture > flower pictures) yielded no significant group differences in the Whole‐brain analysis or the ROI analyses. In addition, there were no instances of greater activation in the control group than the CUD group for any of the unimodal contrasts (Figure 6).

#### 3.5.2 Bimodal cues

**Figure 7:**
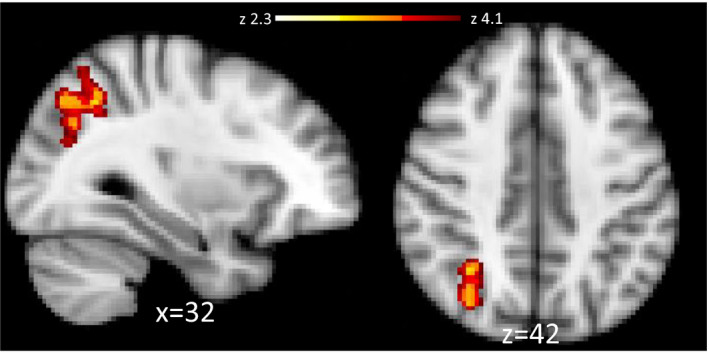
Significantly greater fMRI activation was observed in the CUD > Control contrast in the superior parietal cortex to bimodal cannabis cues with bimodal flower cues as the control condition. Images are presented in radiological convention (R = L). Coordinates are in MNI space. The color bar represents *z* values for voxel within the significant cluster

For the bimodal cannabis cue > baseline contrast, the CUD group showed significantly greater activation in the same regions that were significant in the unimodal cannabis odor > baseline control condition. These regions included the frontal pole, striatum, and bilateral insula. The region of interest analyses yielded significant activation in the left pallidum (*p* = .0229), the VTA (*p* < .0149) but not the nucleus accumbens (*p* > .05; Figure 6). For the bimodal cannabis cue > bimodal flower cue contrast, the CUD group showed increased activation in the superior parietal cortex (Figure 7). There were no significant differences in the ROIs for this contrast. In addition, there were no instances of greater activation in the control group compared to the CUD group. A detailed report of the significant group differences can be found in Table 3.

**Table 3:** Group differences in Whole‐brain fMRI activation to cannabis cues

Contrast	Group diff	Voxels	*p*‐val	*z*‐max	*x* (mm)	*y* (mm)	*z* (mm)	Peak region	Other regions
Cannabis Odor > Baseline	CUD > CON	503	.0412	7.33	14	−22	−22	Brain‐Stem	Ventral Tegmental Area—VTA; Right V; Right I‐IV; Parahippocampal Gyrus—posterior division; Temporal Fusiform Cortex—posterior division; Left Substantia Nigra; Lingual Gyrus; Right Substantia Nigra; Parahippocampal Gyrus—anterior division; Temporal Occipital Fusiform Cortex; Right VI; Inferior Temporal Gyrus—posterior division; Right Hippocampus
	CUD > CON	688	.0074	4.49	24	16	26		Insular Cortex Frontal Orbital Cortex; Frontal Operculum Cortex; Frontal Pole; Inferior Frontal Gyrus—pars triangularis; Right Putamen; Right Caudate
	CUD > CON	705	.0064	5.8	−38	22	0	Frontal Operculum Cortex	Frontal Pole; Frontal Orbital Cortex; Insular Cortex; Left Putamen; Left Caudate; Left Accumbens
Cannabis Picture > Baseline	CUD > CON	556	.0254	7.51	2	−88	20	Cuneal Cortex	Lateral Occipital Cortex—superior division; Lateral Occipital Cortex—inferior division; Occipital Pole
	CUD > CON	574	.0214	4.32	22	20	26		Insular Cortex Frontal Orbital Cortex; Frontal Operculum Cortex; Frontal Pole; Inferior Frontal Gyrus—pars triangularis; Right Putamen; Inferior Frontal Gyrus—pars opercularis; Right Caudate
Cannabis Odor + Picture > Baseline	CUD > CON	548	.0292	5.5	8	−86	32	Cuneal Cortex	Lateral Occipital Cortex—superior division; Lateral Occipital Cortex—inferior division; Occipital Pole
	CUD > CON	618	.0153	6.21	−38	24	0	Frontal Operculum Cortex	Frontal Pole; Frontal Orbital Cortex; Insular Cortex; Left Putamen; Inferior Frontal Gyrus—pars triangularis; Left Caudate; Left Accumbens
Cannabis Bimodal > Flower Bimodal	CUD > CON	525	.0412	3.37	32	−56	42	Superior Parietal Lobule	Lateral Occipital Cortex—superior division; Precuneus Cortex; Angular Gyrus

Note.

R = right, L = left. *x*, *y*, and *z* coordinates are in Montreal Neurological Institute space. "Peak region" and "other regions" are labeled using the Harvard‐Oxford Cortical Structural Atlas, the Harvard‐Oxford Subcortical Structural Atlas, the Cerebellar Atlas, and the Duke Midbrain Atlas. *p*‐values are based on a whole‐brain cluster correction for multiple comparisons.

#### 3.5.3 Correlations with VAS Craving rating in the CUD group

**Figure 8:**
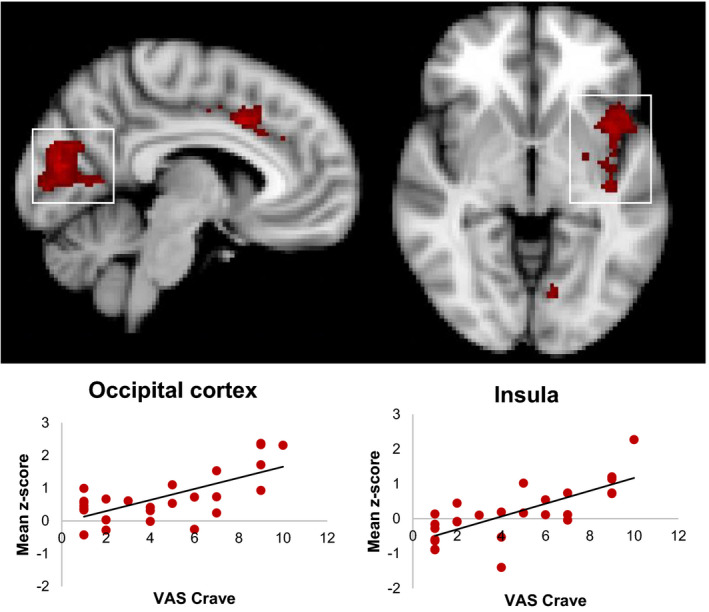
Relationship between fMRI activation to bimodal cannabis cues and self‐reported craving in the CUD group. Clusters signify brain regions showing a significant (*p* < .05, corrected) correlation. Scatter plots depicting the relationship between craving and activation (labeled using a white box) are provided for descriptive purposes only. For each participant, a mean *z*‐score was obtained by averaging the *z*‐score of all the voxels within the mask defined by the significant group cluster and plotted against their VAS craving score

A correlation analysis found significant associations between activation to bimodal cannabis stimulus cues and self‐reported craving measured directly after the fMRI task. For the contrast bimodal cannabis > bimodal flower, higher levels of activation within the cingulate gyrus, left insular cortex, and occipital cortex were associated with higher levels of self‐reported craving following cue exposure (Table 4, Figure 8). There were no significant correlations with the other contrasts.

**Table 4:** fMRI correlations with self‐reported craving

Contrast	+/−	Voxels	*p*‐val	*z*‐max	*x* (mm)	*y* (mm)	*z* (mm)	Peak region	Other regions
Cannabis Bimodal > Flower Bimodal	+	670	.0041	3.95	−2	16	36	Cingulate Gyrus—anterior division	Paracingulate Gyrus; Juxtapositional Lobule Cortex (formerly Supplementary Motor Cortex)
		1,128	<.001	3.45	−46	14	−6	Frontal Operculum Cortex	Insular Cortex; Central Opercular Cortex; Heschl's Gyrus (includes H1 and H2); Planum Polare; Temporal Pole; Left Amygdala; Left Putamen; Postcentral Gyrus; Precentral Gyrus; Parietal Operculum Cortex; Planum Temporale; Frontal Orbital Cortex; Inferior Frontal Gyrus—pars triangularis; Left Caudate; Left Pallidum
		1,840	<.001	3.77	−10	−88	4	Intracalcarine Cortex	Lingual Gyrus; Cuneal Cortex; Supracalcarine Cortex; Precuneus Cortex; Lateral Occipital Cortex—inferior division

Note

R = right, L = left. *x*, *y*, and *z* coordinates are in Montreal Neurological Institute space. "Peak region" and "other regions" are labeled using the Harvard‐Oxford Cortical Structural Atlas, the Harvard‐Oxford Subcortical Structural Atlas, the Cerebellar Atlas, and the Duke Midbrain Atlas. *p*‐values are based on a whole‐brain cluster correction for multiple comparisons.

Because only half of the sample reported using cannabis in the 24‐hr interval following the MRI visit, we did not conduct the planned correlation between fMRI activation to cannabis cues and time interval between the MRI visit and subsequent cannabis use.

## 4 DISCUSSION

This study utilized a new multisensory cannabis cue reactivity paradigm to determine the utility of including odor stimuli in paradigms designed to identify brain regions impacted by problematic cannabis use and associated with self‐reported craving. In addition, we tested whether our experimental paradigm increased self‐reported craving and resulted in cannabis consumption soon after completing the research visit. Participants were screened and excluded for comorbid substance dependence, history of severe psychiatric disorders, and psychotropic medication use to minimize the influence of psychotropic medication or drugs other than cannabis on our brain measures. We found evidence that multisensory cannabis cues activate reward‐related circuitry and are particularly useful for identifying brain regions that are sensitive to individual difference in craving. In addition, although participants reported a significant increase in self‐reported craving following cue exposure, most did not go on to consume cannabis soon after the research visit.

### 4.1 Odor versus picture cues

This study utilized both visual and odor cannabis cues to determine whether cue modality modulated activation within reward circuitry in CUD. To our knowledge, odor stimuli have not been previously utilized in cannabis cue reactivity imaging studies; therefore, the strengths and weaknesses of each stimulus modality for detecting brain changes associated with CUD are unknown. In the contrast identifying brain regions that showed greater activation to the cannabis picture stimuli, we observed significantly greater activation in the entire occipital cortex, the inferior temporal lobes, and the cerebellum. In addition, there was significantly greater activation in the bilateral amygdala, which has been associated with negative emotions and stress (Koob & Volkow, 2016). Notably, the contrast designed to test for greater activation to odor cues compared to picture cues found odor activated brain regions more closely associated with addiction and craving: the right nucleus accumbens, left pallidum, left putamen, and left anterior insular cortex. Additional brain regions showing significantly increased activation to odor cues included the precuneus, supplementary motor area, angular gyrus and superior temporal lobe, and superior frontal gyrus. No significant differences in cue type were observed in the VTA. Our results indicate that odor stimuli may engage reward circuitry to a greater degree than picture stimuli, and thus are a useful addition to cue reactivity paradigms designed to detect neural changes associated with problematic cannabis use.

### 4.2 Brain response to cannabis cues in CUD varies by cue type and baseline

We compared brain activation to cannabis cues to a simple baseline condition (i.e., a fixation cross) and to a closely related stimulus type associated with extrinsic reward: flowers. Flowers are not intrinsically rewarding, like food, but culturally, they are associated with celebrations, holidays, and gifts. They are aesthetically attractive and typically have a pleasant odor. Flowers and tools associated with gardening are visually and semantically related to cannabis and cannabis paraphernalia, except that the flowers depicted in our experiment do not have psychoactive properties. Our use of this stimulus type expands on existing work on cannabis cue reactivity in comparison to other rewarding stimuli such as fruit (see, e.g., Filbey et al., 2016) and sex (Wetherill et al., 2014).

Our comparison of cannabis cues to our simple baseline stimuli in CUD participants yielded widespread robust activation in mesocorticolimbic, insular, cerebellar, parietal and occipital regions to unimodal and bimodal cannabis cues. However, when flower stimuli were the comparison condition, significant activation to cannabis cues was no longer present in several brain regions that are rich in cannabinoid 1 receptors (Parsons & Hurd, 2015) and associated with reward and addiction, namely the VTA, pallidum, and nucleus accumbens. Instead, greater response to cannabis cues was primarily observed in temporooccipital regions associated with object processing and the dorsal attention network.

The contrast comparing bimodal cannabis cues to bimodal flower cues showed significantly greater activation than what was observed with baseline as the control condition. Specifically, activation in additional regions including the OFC, medial prefrontal cortex, anterior cingulate, insula, and amygdala were observed. In addition, this contrast exclusively yielded activation that showed a significant correlation with self‐reported craving in the insula and the anterior cingulate, regions that are putatively associated with addiction‐related preoccupation/anticipation (Koob & Volkow, 2016). Correlations between activation and craving were also observed in the visual cortex, a region that is not typically considered part of reward circuitry, yet is overwhelmingly reported as activated in addiction literature (Charboneau et al., 2013; Hanlon, Dowdle, Naselaris, Canterberry, & Cortese, 2014). It is likely that sensory processing is altered in individuals who are addicted to drugs, via cognitive processes known to impact activity in primary visual cortex (Hanlon et al., 2014). Notably, consistent with the unimodal cue contrasts, activation in the VTA, nucleus accumbens, and pallidum was not significantly higher in response to the cannabis cues relative to the flower stimuli. These findings suggest that the VTA, nucleus accumbens, and pallidum have a generalized hyperresponsivity to pleasant, rewarding stimuli, while other brain regions such as the OFC, amygdala, insula, and anterior cingulate may show a specific enhancement to cannabis cues. These results are partially consistent with work by Wetherill et al. (2014), who found that activation to sexual cues and cannabis cues were similar to each other, and when directly compared, did not result in any significant differences. The authors of this study concluded that chronic drug use did not result in the devaluation of natural rewards (Wetherill et al., 2014). For further information on brain activation to the flower stimuli in the CUD group, see Figure S1 and Table S1.

The results from this study are somewhat inconsistent with Filbey et al.’s study (2016) that used fruit as a natural reward cue. However, it is possible that the reason Filbey et al. found significantly increased activation in mesolimbic cortical regions to cannabis cues compared to fruit was not because enhancement of the mesocorticolimbic reward system is specific to cannabis cues, but because fruit cues do not reliably activate reward circuitry (Goldstone et al., 2009; Mehta et al., 2012) but see (Frasnelli et al., 2015). The results of the contrast comparing fruit cues to neutral object cues found that increased activation to fruit cues was limited to the thalamus, claustrum, and the cerebellum. Similarly, the non‐cannabis‐using controls showed greater activation to fruit cues in the posterior cingulate, superior temporal gyrus, thalamus, and cerebellum. These brain regions are not strongly associated with reward processing. Failure to activate the reward system in the Filbey study may be due to the type of food selected as stimuli. Work done by our group previously showed that neural circuits engaged in reward circuitry are selectively attuned to high‐calorie food that are perceived as fattening (Schur et al., 2009). In our study, fattening food, including candy, desserts, pastries, and high‐fat savory foods such as pizza, hamburgers, chicken wings, and other fried foods showed robust activation in the midbrain, nucleus accumbens, and other regions associated with reward while nonfattening food, including fruits, vegetables, salads, low‐fat meats, and seafood, only showed increased activation in the occipital lobe when compared to neutral objects (Schur et al., 2009). In light of this, future studies comparing food‐based rewards might consider stimuli depicting high‐calorie food as a natural reward.

In support of our a‐priori hypothesis, the CUD group showed increased activation of mesocorticolimbic regions in CUD participants compared to controls. Notably, our CUD participants showed significantly increased neural sensitization in the nigrostriatal pathway, which is a dopaminergic pathway involved in habit formation, and in the VTA and pallidum, which are part of a pathway involved in the reinforcing effects of drugs and relapse (Ahrens, Meyer, Ferguson, Robinson, & Aldridge, 2016; Prasad & McNally, 2016). Regions involved in anticipation of reward and craving, the insula and prefrontal cortex (Koob & Volkow, 2016), also showed significantly higher levels of activation to the cannabis cues compared to our control participants and were correlated to self‐reported craving. Overall, our findings are partially consistent with the incentive sensitization theory, proposing that sensitization of reward circuity from substance abuse generates increased incentive salience of drug cues (Robinson & Berridge, 2008). While we found sensitization of reward circuitry to cannabis cues, this sensitization was also present to our control stimulus, suggesting that sensitization may be generalized across rewarding stimuli.

### 4.3 Limitations

Although our participants were screened for problematic drinking behaviors using the CAGE, many participants in the CUD group still had elevated alcohol use scores on the AUDIT. To better isolate specific cannabis‐related effects, a sample of cannabis‐using individuals who do not consume alcohol would be optimal, albeit atypical. Alternatively, including a second control group that is cannabis‐naïve but is matched on alcohol use (e.g., AUDIT score) to the CUD group would allow us to identify whether cue reactivity to cannabis cues is impacted by comorbid problematic alcohol consumption. A similar approach would also aid in differentiating effects due to cannabis versus tobacco. In addition, education level, socioeconomic status, psychophysiological confirmation of normal olfactory function, and other factors, such as smell sensitivity, that impact olfactory perception were not collected in the present study. Thus, it is possible that observed group differences seen here may be due in part to differences in these potentially confounding variables. Our CUD group did not undergo a clinical diagnostic interview to assess for the presence or absence of Cannabis Use Disorder. Thus, while all participants in the CUD group meet criteria for “at risk” use, this does not preclude them from also meeting DSM‐V criteria for Cannabis Use Disorder. In addition, we did not test the reliability and validity of the post‐fMRI interview questions, which asked participants to report their substance use over the preceding 24 hr. However short‐term recall of substance use using similar measures has been shown to be both reliable and valid in confidential research contexts (Babor, Steinberg, Anton, & Del Boca, 2000; Kypri et al., 2016; Laforge, Borsari, & Baer, 2005; Simons, Wills, Emery, & Marks, 2015) Finally, we did not observe significant group differences when flowers were the baseline condition relative to the cannabis cues. It is possible that an experimental design that increased cognitive expectancies related to cannabis use (e.g., telling participants that the odorant contained THC (see, e.g., Faria et al., 2020) may have elicited a more robust signal to the cannabis cues in reward regions.

## 5 CONCLUSIONS

Our study found that exposure to both visual and odor‐based cannabis cues resulted in significant activation in the neural circuitry involved in craving and reward, specifically the VTA and pallidum and the insula. Although both modalities were sensitive to brain changes associated with problematic cannabis use, a greater neural response was observed to the odor cues in brain regions mediating anticipation and reward, suggesting that cannabis odor stimuli would be a valuable addition to cue reactivity studies.

## CONFLICT OF INTEREST

None declared.

## AUTHOR CONTRIBUTIONS

NK was responsible for the study concept and design. JS assisted with data collection and calculating summary statistics on our behavioral data and questionnaires. FR built the olfactometer and supervised the odor delivery during the fMRI task. BD and BD from the Werc Shop created the cannabis odorant used in the fMRI task. MB and NK performed the imaging analysis. NK and ML assisted with interpretation of findings. NK drafted the manuscript. NK, ML, and FR provided critical revision of the manuscript for important intellectual content. All authors critically reviewed content and approved the final version for publication.

## Supporting information

Supinfo1

Supinfo2
