# Supplementary material for: FMRI activation to cannabis odor cues is altered in individuals at risk for a cannabis use disorder
Source: Brain Behav. 2020 Aug 30;10(10):e01764. doi: 10.1002/brb3.1764 (PMC7559640; doi:10.1002/brb3.1764)

Supplementary Figure 1. Post-hoc analyses of the neural response to flower stimuli were conducted to further understand the negative findings observed when flower stimuli were used as the baseline stimuli in the contrasts. Preprocessing was as described in the manuscript. Additional time series analyses were carried out using FILM with local autocorrelation correction. Condition effects were estimated at each voxel yielding the following contrasts for each participant: flower odor > baseline, flower picture > baseline, and flower odor+picture > baseline. FMRI data were registered to the MPRAGE and then warped to MNI template brain via ANTS diffeomorphic registration.

Higher-level analyses were carried out using FLAME (FSL's Local Analysis of Mixed Effects) stage 1 and stage 2. Whole brain voxelwise comparisons of fMRI activation values in CUD and control participants were performed using t- and F-statistics, which were then converted to *z*-scores by means of a probability integral transformation and thresholded using clusters determined by *z* ≥ 2.3 and a (corrected) cluster significance threshold of *p* < 0.05.

Supplementary Table 1. Group means and differences in whole brain fMRI activation to flower cues

| **Lower-level contrast** | **Higher-level contrast** | **Voxels** | **p-val** | **z-max** | **x (mm)** | **y (mm)** | **z (mm)** | **Peak Region** | **Other regions** |
| --- | --- | --- | --- | --- | --- | --- | --- | --- | --- |
| Flower Odor > Baseline | CON | 2596 | < .001 | 6.35 | -4 | -8 | -8 | Left Substantia Nigra | Brain-Stem; Ventral Tegmental Area - VTA; Right I-IV; Left VI; Left Thalamus; Right Thalamus; Left I-IV; Left V; Right V; Vermis VIIIa; Vermis VI; Right VI; Left VIIb; Vermis VIIIb; Vermis IX; Vermis VIIb; Left VIIIa; Vermis X; Right Substantia Nigra; Vermis Crus II; Left Crus II; Right VIIb; Left VIIIb; Right VIIIa; Left IX; Right Crus II; Right IX; Left Crus I |
|  |  | 4833 | < .001 | 7.12 | 16 | -92 | -12 | Occipital Pole | Right Crus I; Right Crus II; Lateral Occipital Cortex - superior division; Lateral Occipital Cortex - inferior division; Angular Gyrus; Right VI; Vermis VI |
|  |  | 10294 | < .001 | 6.44 | -32 | -6 | -2 | Left Putamen | Frontal Pole; Paracingulate Gyrus; Right Putamen; Precentral Gyrus; Cingulate Gyrus - anterior division; Central Opercular Cortex; Inferior Frontal Gyrus - pars opercularis; Inferior Frontal Gyrus - pars triangularis; Insular Cortex; Left Hippocampus; Left Amygdala; Right Pallidum; Frontal Medial Cortex; Frontal Operculum Cortex; Frontal Orbital Cortex; Superior Frontal Gyrus; Middle Frontal Gyrus; Right Amygdala; Left Pallidum; Right Accumbens |
|  | CUD | 5976 | < .001 | 9.03 | -56 | -64 | 24 | Lateral Occipital Cortex - superior division | Occipital Pole; Lateral Occipital Cortex - inferior division; Angular Gyrus; Supramarginal Gyrus - posterior division |
|  |  | 26741 | < .001 | 14 | 0 | -56 | -18 | Left I-IV | Ventral Tegmental Area - VTA; Left VI; Frontal Pole; Right V; Left V; Right I-IV; Right VI; Left Crus I; Right Crus I; Left Substantia Nigra; Right Substantia Nigra; Vermis VI; Paracingulate Gyrus; Brain-Stem; Insular Cortex; Left Crus II; Right Putamen; Right Crus II; Central Opercular Cortex; Left Putamen; Cingulate Gyrus - anterior division; Vermis VIIIa; Temporal Occipital Fusiform Cortex; Lingual Gyrus; Inferior Frontal Gyrus - pars opercularis; Precentral Gyrus; Left Hippocampus; Frontal Operculum Cortex; Inferior Frontal Gyrus - pars triangularis; Right Thalamus; Temporal Fusiform Cortex - posterior division; Left Amygdala; Supramarginal Gyrus - anterior division; Vermis Crus II; Vermis IX; Left Thalamus; Vermis VIIIb; Parahippocampal Gyrus - posterior division; Left Pallidum; Frontal Orbital Cortex; Left VIIb; Vermis X; Vermis VIIb; Left Caudate; Right Pallidum; Right Caudate; Left VIIIa; Left Accumbens; Left VIIIb; Right Accumbens; Left IX; Right VIIb; Right Amygdala; Vermis Crus I; Right VIIIa; Right IX |
|  | CUD>CON | 641 | .011 | 6.36 | -40 | 18 | -12 | Frontal Orbital Cortex | Frontal Pole; Insular Cortex; Left Putamen; Frontal Operculum Cortex; Left Caudate; Left Accumbens |
|  |  | 680 | .008 | 4.69 | 44 | 16 | -6 | Insular Cortex | Frontal Orbital Cortex; Frontal Operculum Cortex; Frontal Pole; Inferior Frontal Gyrus - pars triangularis; Right Putamen; Inferior Frontal Gyrus - pars opercularis; Right Caudate |
| Flower Picture > Baseline | CON | 2431 | < .001 | 7.2 | 2 | -10 | -10 | Ventral Tegmental Area - VTA | Brain-Stem; Right I-IV; Left Thalamus; Left VI; Right Thalamus; Left I-IV; Left V; Right V; Vermis VIIIa; Vermis VI; Right VI; Vermis VIIIb; Left VIIb; Left VIIIa; Vermis VIIb; Right Substantia Nigra; Left Crus II; Vermis Crus II; Left Substantia Nigra; Vermis IX; Right VIIb; Left VIIIb; Right VIIIa; Left Crus I; Right Crus II; Right IX |
|  |  | 6419 | < .001 | 8.63 | 10 | -94 | 6 | Occipital Pole | Right Crus I; Left Crus I; Left Crus II; Right Crus II; Lateral Occipital Cortex - superior division; Lateral Occipital Cortex - inferior division; Lingual Gyrus; Occipital Fusiform Gyrus; Intracalcarine Cortex; Left VI; Right VI; Vermis VI; Left VIIb |
|  |  | 9192 | < .001 | 6.31 | 34 | 36 | -8 | Frontal Pole | Paracingulate Gyrus; Right Putamen; Precentral Gyrus; Cingulate Gyrus - anterior division; Central Opercular Cortex; Inferior Frontal Gyrus - pars opercularis; Inferior Frontal Gyrus - pars triangularis; Insular Cortex; Right Pallidum; Frontal Medial Cortex; Frontal Orbital Cortex; Superior Frontal Gyrus; Middle Frontal Gyrus; Right Amygdala; Right Accumbens |
|  | CUD | 35291 | < .001 | 14.2 | -2 | -58 | -22 | Left V | Ventral Tegmental Area - VTA; Left VI; Right V; Right I-IV; Frontal Pole; Left I-IV; Right VI; Right Crus I; Left Crus I; Left Substantia Nigra; Occipital Pole; Right Substantia Nigra; Vermis VI; Lateral Occipital Cortex - superior division; Brain-Stem; Paracingulate Gyrus; Right Crus II; Lateral Occipital Cortex - inferior division; Left Crus II; Lingual Gyrus; Insular Cortex; Vermis VIIIa; Right Putamen; Left Putamen; Central Opercular Cortex; Temporal Occipital Fusiform Cortex; Cingulate Gyrus - anterior division; Left Hippocampus; Vermis IX; Inferior Frontal Gyrus - pars opercularis; Precentral Gyrus; Vermis Crus II; Inferior Frontal Gyrus - pars triangularis; Frontal Operculum Cortex; Vermis VIIIb; Left Amygdala; Right Thalamus; Temporal Fusiform Cortex - posterior division; Left Thalamus; Parahippocampal Gyrus - posterior division; Vermis X; Left Pallidum; Left VIIb; Occipital Fusiform Gyrus; Frontal Orbital Cortex; Vermis VIIb; Left Caudate; Left VIIIa; Right Pallidum; Right VIIb; Right Caudate; Left VIIIb; Left Accumbens; Right IX; Left IX; Vermis Crus I; Right Accumbens; Right VIIIa; Right Amygdala |
|  | CUD>CON | 681 | < .001 | 6.08 | -32 | 38 | 10 | Frontal Pole | Frontal Orbital Cortex; Insular Cortex; Left Putamen; Frontal Operculum Cortex; Left Caudate; Left Accumbens |
|  |  | 729 | .005 | 5.66 | 46 | 20 | 0 | Frontal Operculum Cortex | Insular Cortex; Frontal Orbital Cortex; Frontal Pole; Inferior Frontal Gyrus - pars triangularis; Paracingulate Gyrus; Right Putamen; Inferior Frontal Gyrus - pars opercularis; Right Caudate |
| Flower Bimodal > Baseline | CON | 2328 | < .001 | 6.17 | 0 | -6 | -6 | Left Thalamus | Brain-Stem; Ventral Tegmental Area - VTA; Right I-IV; Right Thalamus; Left VI; Left I-IV; Left V; Right V; Vermis VIIIa; Vermis VI; Right VI; Left VIIb; Vermis VIIIb; Left VIIIa; Vermis VIIb; Right Substantia Nigra; Left Crus II; Vermis Crus II; Right VIIb; Left VIIIb; Left Substantia Nigra; Vermis IX; Right VIIIa; Right Crus II; Left Crus I; Right IX |
|  |  | 6196 | < .001 | 7.21 | 26 | -76 | -10 | Occipital Fusiform Gyrus | Right Crus I; Left Crus I; Occipital Pole; Left Crus II; Right Crus II; Lateral Occipital Cortex - superior division; Lateral Occipital Cortex - inferior division; Lingual Gyrus; Left VI; Right VI; Vermis VI; Left VIIb |
|  |  | 9367 | < .001 | 7.69 | 28 | -10 | -4 | Right Putamen | Frontal Pole; Paracingulate Gyrus; Cingulate Gyrus - anterior division; Precentral Gyrus; Inferior Frontal Gyrus - pars opercularis; Central Opercular Cortex; Inferior Frontal Gyrus - pars triangularis; Insular Cortex; Left Putamen; Left Hippocampus; Left Amygdala; Right Pallidum; Frontal Orbital Cortex; Frontal Medial Cortex; Superior Frontal Gyrus; Middle Frontal Gyrus; Right Amygdala; Left Pallidum; Right Accumbens |
|  | CUD | 34882 | < .001 | 13.3 | -2 | -56 | -18 | Left V | Ventral Tegmental Area - VTA; Left VI; Right V; Right I-IV; Frontal Pole; Right VI; Left I-IV; Left Crus I; Right Crus I; Left Substantia Nigra; Occipital Pole; Right Substantia Nigra; Vermis VI; Lateral Occipital Cortex - superior division; Lateral Occipital Cortex - inferior division; Brain-Stem; Right Crus II; Left Crus II; Paracingulate Gyrus; Insular Cortex; Right Putamen; Vermis VIIIa; Lingual Gyrus; Left Putamen; Central Opercular Cortex; Temporal Occipital Fusiform Cortex; Cingulate Gyrus - anterior division; Left Hippocampus; Inferior Frontal Gyrus - pars opercularis; Precentral Gyrus; Vermis Crus II; Vermis IX; Right Thalamus; Inferior Frontal Gyrus - pars triangularis; Vermis VIIIb; Frontal Operculum Cortex; Temporal Fusiform Cortex - posterior division; Left Amygdala; Left Thalamus; Parahippocampal Gyrus - posterior division; Left Pallidum; Left VIIb; Occipital Fusiform Gyrus; Frontal Orbital Cortex; Vermis X; Vermis VIIb; Left VIIIa; Right Pallidum; Left Caudate; Right VIIb; Left VIIIb; Left Accumbens; Right Amygdala; Vermis Crus I; Left IX; Right Caudate; Right VIIIa; Right Accumbens; Right IX; Right Hippocampus |
|  | CUD>CON | 605 | .017 | 5.55 | 42 | 22 | 2 | Frontal Operculum Cortex | Insular Cortex; Frontal Orbital Cortex; Frontal Pole; Inferior Frontal Gyrus - pars triangularis; Middle Frontal Gyrus; Right Putamen; Right Caudate |
|  |  | 691 | .008 | 6.19 | -40 | 18 | -8 | Insular Cortex | Frontal Pole; Frontal Orbital Cortex; Left Putamen; Frontal Operculum Cortex; Left Caudate; Left Accumbens |

Note. R = right, L = left. X, y, and z coordinates are in Montreal Neurological Institute space. "Peak region" and "other regions" are labeled using the Harvard-Oxford Cortical Structural Atlas, the Harvard-Oxford Subcortical Structural Atlas, the Cerebellar Atlas, and the Duke Midbrain Atlas. P-values are based on a whole-brain cluster correction for multiple comparisons.

Supplementary Figure 1. Significant between group difference (CUD>CON) in fMRI activation to unimodal and bimodal flower cues compared to Baseline. The clusters depicted reflect significantly greater activation in the CUD group based on p < .05, whole brain correction. Bar graphs show group means for the CON group (blue) and the CUD group (red) for the pallidum (left) and VTA (right). Images are presented in radiological convention (R=L)


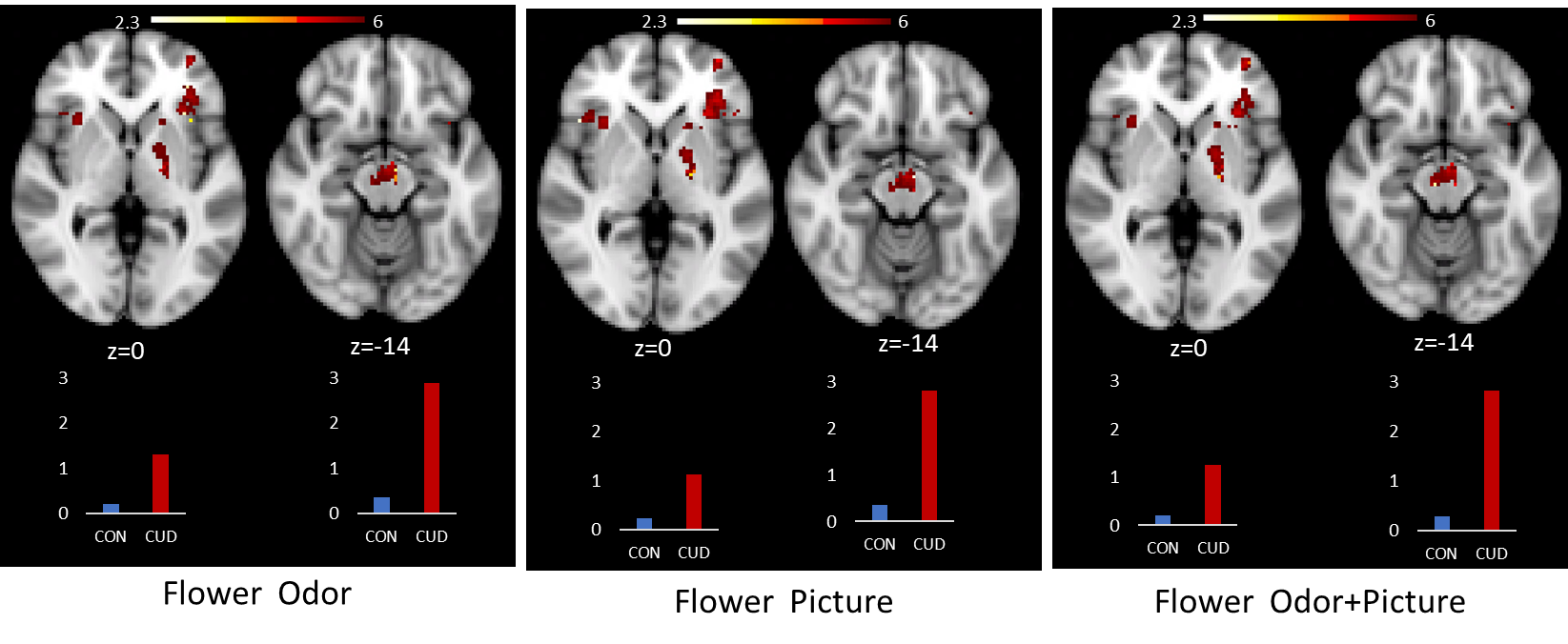

Supplement: Supplementary file 1 — Supinfo1 [file BRB3-10-e01764-s001.docx]
