# Supplementary material for: FMRI activation to cannabis odor cues is altered in individuals at risk for a cannabis use disorder
Source: Brain Behav. 2020 Aug 30;10(10):e01764. doi: 10.1002/brb3.1764 (PMC7559640; doi:10.1002/brb3.1764)

# SUPPLEMENTAL INFORMATION

Part 1. Lifetime Use of Other Substances

During the semi-structured screening phone call, participants were asked about any lifetime use of illicit substances other than cannabis, alcohol and tobacco. Specifically, they were asked about use of the following:

- Cocaine (coke, crack, etc.),
- Prescription stimulants (Ritalin, Concerta, Dexedrine, Adderall, diet pills, etc.),
- Methamphetamine (speed, crystal meth, ice, etc.),
- Inhalants (nitrous oxide, glue, gas, paint thinner, etc.),
- Sedatives or sleeping pills (Valium, Serepax, Ativan, Xanax, Librium, Rohypnol, GHB, etc.),
- Hallucinogens (LSD, acid, mushrooms, PCP, Special K, ecstasy, etc.),
- Street opioids (heroin, opium, etc.),
- Prescription opioids (fentanyl, oxycodone/OxyContin/Percocet, hydrocodone/Vicodin, methadone, buprenorphine, etc.).

The ASSIST screening questions were administered for all substances that a participant reported using at any point in their lifetime. Individuals receiving an ASSIST score ≥ 4 for any of the above substances were not enrolled in the study. Thus, all enrolled participants had either an ASSIST score ≤3, indicating limited lifetime use with no functional impairments, or no ASSIST score, due to no lifetime use of that substance (Table S1).


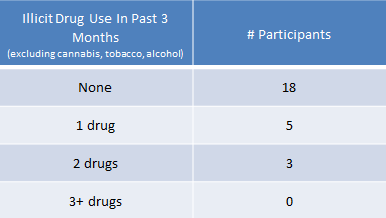

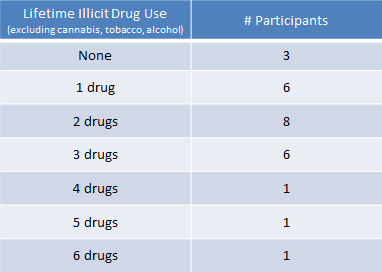


Table S1. Use of other substances (excludes cannabis, tobacco, and alcohol) by participants in CUD group over (a) lifetime and (b) in past three months.

a)

b)

Table S2 shows a tally of the number of CUD participants who received a score of 0, 2, or 3 on the ASSIST, organized by substance. For this sample, an ASSIST score of 0 indicates at least one instance of use in the individual’s lifetime, but no use in the past three months and no symptoms of problematic use or functional impairment. An ASSIST score of 2 indicates use of the substance once or twice in the past three months but with no symptoms of problematic use or functional impairment. The score of 3 for Inhalants below was received due to a prior lifetime use and concern from a friend/relative but no use in the past three months and no other symptoms of problematic use or functional impairment. A score of 1 on this measure is not possible.


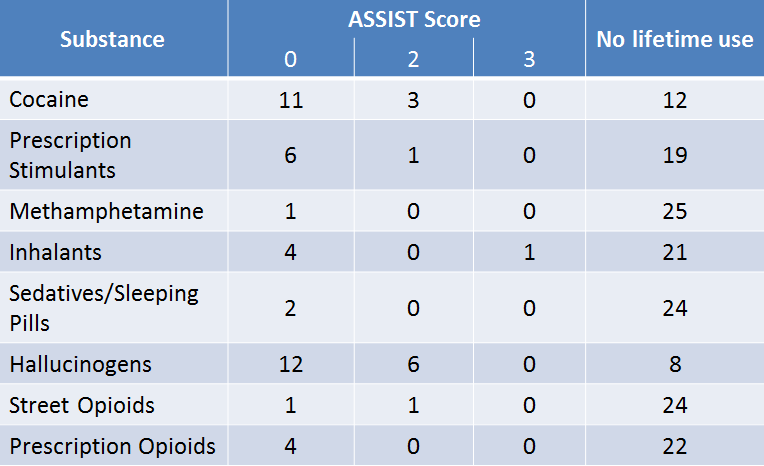


Table S2. Use of substances other than cannabis, alcohol, and tobacco in the CUD group. ASSIST scores of 0-3 indicate low risk of substance use disorder, a determination based on history of use and presence/absence of functional impairments.

Only one individual in the control group (1/25) reported using a substance other than alcohol or tobacco over their lifetime. This control participant reported a single instance of hallucinogen use (ecstasy) but no use in the past three months and no symptoms of problematic use or functional impairment. This participant is not included in the above tables.

Part 2. Cannabis Use Patterns in CUD Group

The cannabis use patterns of our CUD group were identified using a self-report questionnaire developed in our laboratory. Our frequency estimate was obtained from the CUDIT. Using the responses obtained through these measures, we compiled the following data regarding frequency of cannabis use, mode of use, amounts of use, and strain preferences. In addition to the descriptive data reported in Table 1 of the manuscript, the below graphics provide more detail into how the CUD participants in this study are using cannabis.


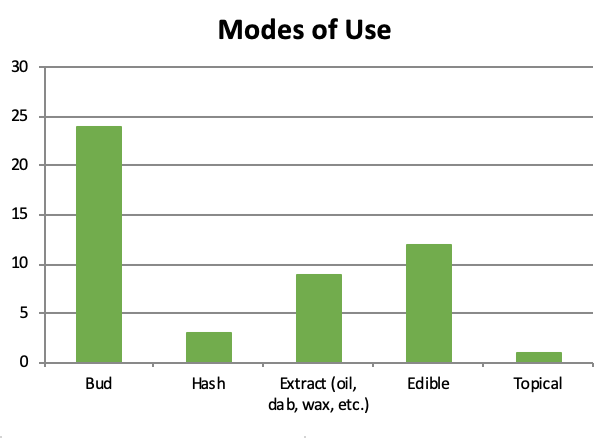
Eighty-one percent of CUD participants report using cannabis at least twice a week in the past 6 months (Figure S1). This group of participants used several different forms of cannabis product (Figures S2 & S3). All but one used some form of “bud” cannabis, which includes use in a joint, blunt, spliff, pipe, bong, or vaporizer. Approximately two-thirds of participants (69%) use multiple forms of cannabis product, usually some combination of “bud”, concentrated extracts, and edibles (Figure S3).

Figure S2. Modes of cannabis use in the CUD group. Participants were asked to select the forms of cannabis that they typically use. Number of participants refers to the total number of participants reporting each mode of use. Thus, participants are counted multiple times if they report multiple modes of use. **Bud** includes consumption via joint, blunt, spliff, pipe, bong, and vaporizer. **Edible** includes swallowed products and tinctures absorbed under the tongue.


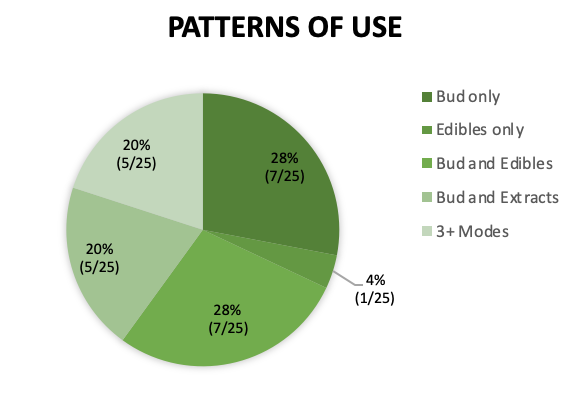


Figure S3. Use of single versus multiple types of cannabis product in the CUD group. Each participant is counted exactly once. **Extract** includes concentrated oil, dab, wax, shatter, and kief (crystals).

Because quantities and scales differ based on form of cannabis used, participants were asked separately for amount consumed in a typical day for “bud” forms of cannabis (in grams) and for edible forms of cannabis. Commercially-produced edibles are dosed separately for the primary psychoactive cannabinoids, Δ^9^-tetrahydrocannabinol (THC) and cannabidiol (CBD), so participants were asked to report the amount in (milligrams) of both THC and CBD. For participants reporting a range of quantities (e.g. 0.1-0.2 grams), we used the mean of the two values provided. A total of 24 participants reported using “bud” forms of cannabis and of these, 20 provided an approximate amount consumed on a typical day of use. Four participants reported using “< 1 gram”, which we determined was insufficiently precise to include in our calculations. On a typical day of use, “bud” users reported consuming an average of 0.376 grams (*SD*= 0.31, range: 0.05 g – 1.0 g) (Figure S4). Among the 14 participants reporting use of edibles, 10 reported THC amounts and 5 reported CBD amounts. On a typical day, these edible users reported consuming an average of 18.32 mg of THC (*SD* = 13.35, range: 4.0 mg – 40 mg) and 10.50 mg CBD (*SD* = 2.74, range: 7.5 mg – 15 mg) (Figure S5).


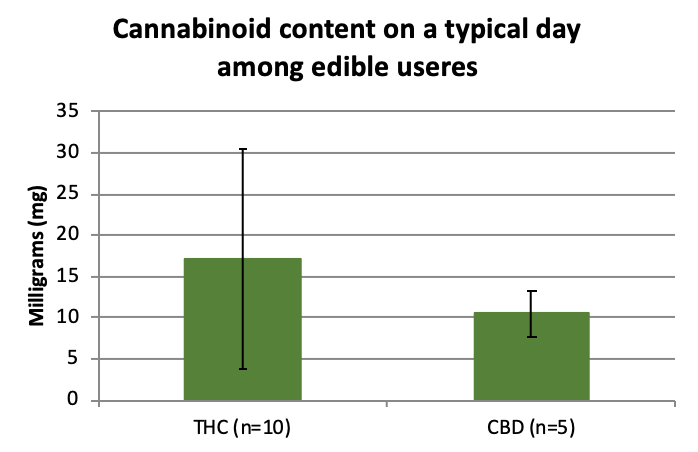


Figure S5. Typical amounts of THC and CBD consumed per day by participants who reported using edible forms of cannabis

Figure S4. Typical amount of cannabis consumed per day by participants who reported using “bud” forms of cannabis, in grams. Bars indicate standard deviations.


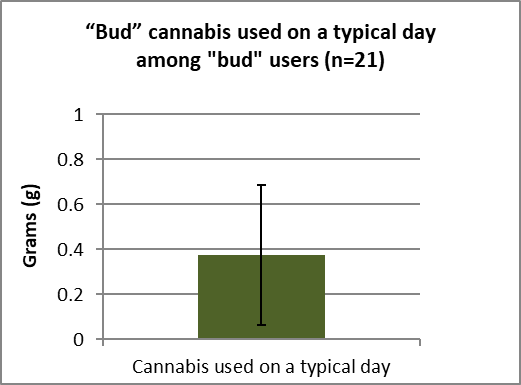


Extract forms of cannabis (oils, wax, crystals, etc.) are typically more potent than other forms, with THC concentrations upward of 80-90%, and are therefore often used in much smaller quantities that are difficult to measure. However, recreational cannabis shops usually sell these forms in standard units of either 0.5 grams or 1.0 grams. Thus, to assess quantity of extract products consumed, participants who reported using extracts were asked how long one-half gram of concentrate usually lasts them. The responses varied widely, from 4 hours to 2 months (*M* = 17.57 days, *SD* = 19.80) (Figure S6). It is important to note that all participants using
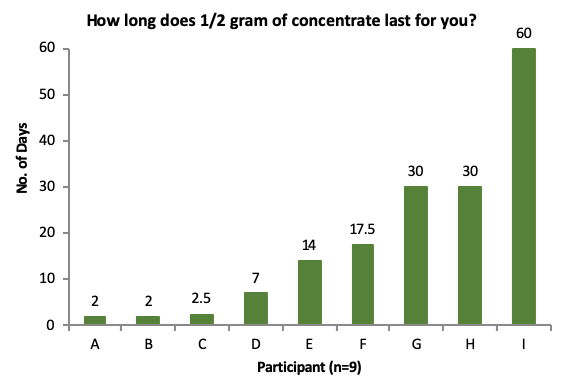
concentrated cannabis products also reported using other forms of cannabis, including bud and edibles, so their cannabis use was likely much greater in the reported time span than one half gram.


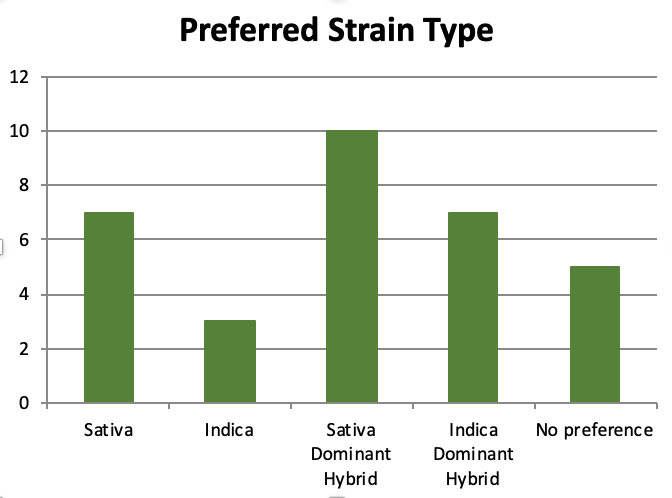
Furthermore, participants provided information regarding their preferred strain types (Figure S7). Our CUD sample shows a greater preference for sativa-type strains over indica strains, although one-third had no strong preference in either direction. Cannabis vendors often classify their products as either sativa, indica, or a hybrid of multiple strains, depending on the source plant and cannabinoid content. Within cannabis-using communities, sativa-dominant strains are characterized as having a higher THC content and lower CBD content, producing a more energizing “head high”. In contrast, indica is more often used in reference to strains with higher CBD and lower THC concentrations, resulting in a more relaxing “body high” with analgesic and nausea-reducing effects. While these characterizations have not been supported by scientific research, cannabis users still seek out different strains based on these purported properties and effects.  ^1^

^1^Rahn, Bailey. (2018, September 30). Indica vs. Sativa: An Overview of Cannabis Types. *Leafly*. Retrieved from https://www.leafly.com/

Figure S6. Nine participants reporting use of cannabis extract products were asked how long ½ gram of product typically lasts for them. Each bar in this figure represents a different individual.

Figure S7. Participant preference for strain type. A) Sativa dominant versus indica dominant strains. Nearly half of CUD participants prefer pure sativa strains and/or sativa dominant hybrids. B) Preferred strain types separated out into sativa versus indica and pure versus hybrid forms.


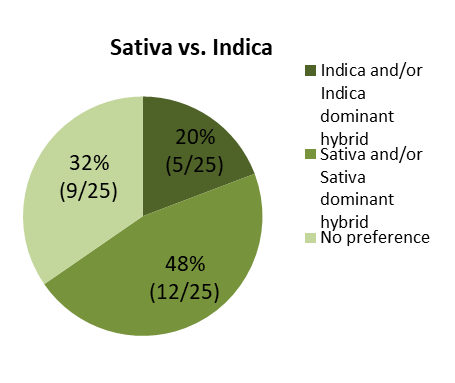

Supplement: Supplementary file 2 — Supinfo2 [file BRB3-10-e01764-s002.docx]
